# Photosynthetic acclimation to warming in tropical forest tree seedlings

**DOI:** 10.1093/jxb/erx071

**Published:** 2017-04-27

**Authors:** Martijn Slot, Klaus Winter

**Affiliations:** 1 Smithsonian Tropical Research Institute, Apartado 0843-03092, Balboa, Republic of Panama

**Keywords:** Carbon uptake, climate change, dark respiration, global warming, *J*_Max_, photosynthesis, temperature-response curve, thermal acclimation, tropical forest, *V*_CMax_.

## Abstract

Tropical forests have a mitigating effect on man-made climate change by acting as a carbon sink. For that effect to continue, tropical trees will have to acclimate to rising temperatures, but it is currently unknown whether they have this capacity. We grew seedlings of three tropical tree species over a range of temperature regimes (*T*_Growth_ = 25, 30, 35 °C) and measured the temperature response of photosynthetic CO_2_ uptake. All species showed signs of acclimation: the temperature-response curves shifted, such that the temperature at which photosynthesis peaked (*T*_Opt_) increased with increasing *T*_Growth_. However, although *T*_Opt_ shifted, it did not reach *T*_Growth_ at high temperature, and this difference between *T*_Opt_ and *T*_Growth_ increased with increasing *T*_Growth_, indicating that plants were operating at supra-optimal temperatures for photosynthesis when grown at high temperatures. The high-temperature CO_2_ compensation point did not increase with *T*_Growth_. Hence, temperature-response curves narrowed with increasing *T*_Growth_. *T*_Opt_ correlated with the ratio of the RuBP regeneration capacity over the RuBP carboxylation capacity, suggesting that at high *T*_Growth_ photosynthetic electron transport rate associated with RuBP regeneration had greater control over net photosynthesis. The results show that although photosynthesis of tropical trees can acclimate to moderate warming, carbon gain decreases with more severe warming.

## Highlight

Seedlings of tropical tree species can acclimate to moderate warming, thereby improving their photosynthetic carbon uptake, but with further warming carbon uptake decreases as plants increasingly operate at supra-optimal temperatures.

## Introduction

Green plants currently remove 123 Gt carbon from the atmosphere every year through photosynthesis (IPCC, 2014), and thereby help to mitigate the rise in CO_2_ levels associated with anthropogenic activity. However, soil and plant respiration combined cause an annual efflux of CO_2_ from the terrestrial biosphere of similar magnitude, ~119 Gt (IPCC, 2014). Carbon sequestration of the terrestrial biosphere thus reflects a precarious balance between two very large fluxes, both of which are sensitive to changes in environmental conditions. Tropical forests play a particularly large role (Stephens *et al.*, 2007; Schimel *et al.*, 2015), with intact tropical forests currently accounting for an annual net carbon uptake of ~2.3 Gt (Pan *et al.*, 2011). A warming-induced reduction in net carbon uptake by plants could lead to a positive feedback between the terrestrial biosphere and the atmosphere.

In the short term, photosynthesis, like any other metabolic process, is sensitive to changes in temperature. Its temperature response tends to follow a peaked curve, where increasing the temperature initially stimulates photosynthesis up to the leaf’s optimum temperature, beyond which photosynthesis declines for biochemical and hydraulic reasons (Sage and Kubien, 2007), until net photosynthesis reaches zero at the high-temperature CO_2_ compensation point. Warming in cool climates may be favorable for CO_2_ uptake (Keenan *et al.*, 2014), but this may not apply to tropical forests, which are already the warmest closed-canopy ecosystem on earth (Wright *et al.*, 2009). While warming leads to accelerated plant growth in most climate zones, in the tropics warming is believed to decrease growth (Lin *et al.*, 2010; Way and Oren, 2010), and the response of photosynthesis may similarly be negative in the tropics if warming causes plants to operate photosynthetically at supra-optimal temperatures.

Plants may acclimate to changes in temperature regime, which results in an improvement of performance at the new, warmer conditions, relative to the performance of unacclimated plants (Berry and Björkman, 1980). In the case of photosynthesis, acclimation typically results in a shift of the temperature-response curve, such that the optimum temperature for photosynthesis of the acclimated plants approaches the new temperature (Way and Yamori, 2014;Slot and Winter, 2016). The shift in optimum temperature may result from increasing control of light-saturated photosynthesis by electron transport capacity (the maximum rate of RuBP regeneration) relative to Rubisco activity (the maximum rate of RuBP carboxylation), the two of which differ in their temperature optima (e.g. Hikosaka *et al.*, 2006). As the temperature-response curves shift, photosynthesis at the optimum temperature may increase, decrease, or stay the same, and as a result, photosynthesis at mean growth temperature – of critical importance for plant performance – may change even if the optimum temperature for photosynthesis is perfectly adjusted to the new growth temperature (Way and Yamori, 2014;Slot and Winter, 2016).

It has long been thought that tropical species may have very limited capacity for thermal acclimation, because in the absence of seasonal temperature changes the selective pressure for thermal acclimation capacity may have been weak (Janzen, 1967). When Doughty (2011) warmed leaves of tropical trees and lianas (woody vines), systematic decreases in photosynthesis suggested that these leaves failed to acclimate. The reduced performance was, however, thought to have resulted from leaf damage caused by occasionally extreme leaf temperatures rather than from a change in average temperature, and because photosynthesis was only measured at two temperatures, acclimation – or the lack thereof – could not easily be inferred from these data. Cheesman and Winter (2013) measured photosynthesis in relation to temperature for tropical tree seedlings grown at sub-optimal and supra-optimal temperatures and found species-specific patterns in the responses. However, in the absence of full temperature-response curves, acclimation could not easily be characterized. Kositsup *et al.* (2009) reported photosynthesis–temperature response curve data for *Hevea brasiliensis*, a tropical tree species cultivated for the production of natural rubber. They demonstrated that photosynthesis acclimated to a reduction in ambient temperature (18 versus 28 °C), as indicated by a lowering of the optimum temperature of photosynthesis. Cunningham and Read (2002) showed that tropical tree species from Australia grown at a temperate temperature regime did more poorly than temperate trees grown at tropical conditions, and that tropical species had narrower temperature ranges over which they could operate at 80% or more of their photosynthetic capacity. Although these results show that some tropical species have greater physiological plasticity than others, in the context of global warming it needs to be examined whether photosynthesis can acclimate to warming above current temperatures in the tropics, rather than whether it can acclimate to lower temperatures.

Lack of such data and of information on the potential feedbacks between the tropical terrestrial biosphere and the atmosphere are major impediments to reducing uncertainty in predictions of coupled climate–vegetation models (e.g. Ahlström *et al.*, 2012; Lombardozzi *et al.*, 2015). In the study presented here, we address the following questions. First, can photosynthesis of tropical tree species acclimate to elevated growth temperature? And second, if so, how does acclimation manifest itself? To answer these questions, we cultivated seedlings of three tropical tree species – the pioneers *Ficus insipida* and *Ochroma pyramidale*, and the late-successional species *Calophyllum longifolium* – at three different temperatures, measured growth, and determined the temperature response of light-saturated photosynthesis and the maximum rates of RuBP carboxylation and regeneration. We hypothesized that warming would result in a shift of key photosynthetic parameters, including the optimal temperature of net photosynthesis, and in adjustments of the maximum rates of RuBP carboxylation and RuBP regeneration, and their ratio.

## Materials and methods

### Plant material and growth conditions

We selected three widely distributed Neotropical tree species that differ in their juvenile light requirements and growth rates. *Ficus insipida* Willd. (Moraceae) is a fast-growing, light-demanding species that regenerates in forest gaps and occurs predominantly in moist forests in Panama, where it is most common in comparatively young stands. *Ochroma pyramidale* (Cav. ex Lam.) Urb. (Malvaceae) is also a fast-growing, light-demanding species that is common in disturbed areas, with its distribution in Panama ranging from tropical dry-deciduous forests to evergreen wet forests and pre-montane forests. *Calophyllum longifolium* L. (Calophyllaceae) is a comparatively slow-growing, shade-tolerant species that is widespread in moist, wet, and lower-montane forests in Panama. Seeds were collected near Panama City, Republic of Panama (mean annual temperature ~27.0 °C), and germinated in trays with potting soil (Miracle-Gro®, Stern’s Miracle-Gro Products, Port Washington, NY). After germination, seedlings were transplanted to individual 1.7-l pots (Short One Treepot™, Stuewe and Sons, Tangent, Oregon) and randomly assigned to one of three growth cabinets (Environmental Growth Chambers, Chagrin Falls, OH, USA) set to 25/25 °C, 30/30 °C and 35/35 °C day/night. Because it is unclear to which component of the temperature regime plants acclimate (e.g. do they acclimate to minimum, maximum, or mean temperatures?), we maintained the same temperatures day and night. At the start of the experiment photosynthetically active radiation at plant height was 450 µmol m^–2^ s^–1^; day length was set to 12 h. Relative humidity during the day was ~66, 55, and 42% in the 25, 30, and 35 °C chambers, respectively. *Calophyllum longifolium* plants at 35 °C did not produce new leaves large enough for gas-exchange measurements. A second batch of seedlings similarly failed to develop measurable leaves. Therefore, a new group of seedlings was placed in a 33/33 °C growth chamber and leaves developed in this chamber were subsequently used for gas-exchange measurements. *Calophyllum longifolium* plants grown at 35 °C were included in the analysis of plant growth (see below).

### Temperature response of net photosynthesis

After the seedlings were exposed to experimental temperature regimes for at least 6 weeks, the temperature response of net photosynthesis was measured between 20 and 55 °C on attached leaves (*n*=4–6; one leaf per plant) that had developed in the experimental treatment, in a temperature-controlled cuvette (GWK-3M; Walz GmbH, Eiffeltrich, Germany) that formed part of an open gas-exchange system composed of other Walz-components and an LI-6252 infrared gas analyzer (LI-COR Biosciences, Lincoln, Nebraska, USA). Leaves were sealed in the chamber at the petiole, enabling measurements of entire leaves. A red-blue LED grow panel (SS-GU300w, Sunshine Systems, Wheeling, IL, USA) placed ~30 cm above the cuvette illuminated the leaf with 1000 µmol m^–2^ s^–1^. Measurements were made at a CO_2_ concentration of ~400 ppm. Leaf temperature (*T*_Leaf_) was monitored abaxially with an OS36T infrared thermocouple (Omega, Stamfort, CT, USA). Spot measurements with a copper–constantan fine-wire thermocouple yielded identical results. Net photosynthesis was first measured at 25 °C, after which a complete temperature response curve was determined between 20 and 55 °C with 5 °C incremental steps and full equilibration at each temperature (15–60 min). Whenever the second reading at 25 °C deviated >10% from the first, measurements were terminated and the data discarded. Along with photosynthesis, the dew-point temperatures of the air entering the cuvette and that of the air exiting were recorded at each temperature. From this, transpiration rates, stomatal conductance, and the leaf-to-air vapor pressure deficit (VPD) were calculated. Leaf area was determined with an LI-3100 leaf area meter (LI-COR), and gas-exchange rates were expressed per unit leaf area. Oven-dried leaves were ground to a fine powder and leaf carbon and nitrogen (N) content were determined by automated combustion and thermal conductivity detection using a Thermo Flash EA1112 analyzer (Waltham, MA, USA).

Temperature response data were fitted following Cunningham and Read (2002) as:

$$\text{Net photosynthesis}\;=\;b\;\times \;\left(T_{Leaf}-T_{Min}\right)\;\times \;\left(1-e^{\text{c}\left(T_{Leaf}-T_{Max}\right)}\right)$$ (1)

Where *T*_Leaf_ is the leaf temperature, *T*_Min_ and *T*_Max_ are the hypothetical low- and high-temperature CO_2_ compensation points, and b and c are constants; these variables were all estimated simultaneously using a non-linear solver function. These curves generally fitted the data well, but they tend to underestimate the temperature optimum (*T*_Opt_) when there are few data points below the peak of the curve, which is particularly common for plants grown at 25 °C. To verify whether Eqn. 1 led to systematic underestimation of *T*_Opt_, we also fitted the data according to June *et al.* (2004) as:

$$\text{Net photosynthesis}\;=\;P_{Opt}\;\times \;e^{–{\left(\frac{T_{Leaf\;}–\;T_{Opt}}{\Omega }\right)}^{2}}$$ (2)

where *Ω* is the curve’s ‘steepness’ parameter that represents the difference in temperature between *T*_Opt_ and the temperature at which photosynthesis (*P*) drops to *e*^–1^ (37%) of its value at *T*_Opt_. These curves were fitted using the non-linear least-squares function ‘nls’ in the ‘stats’ package in R version 3.1.3 (www.r-project.org), and standard errors were determined for all parameters.

### A-c_i_*curves*

When plants had developed at least three new leaves during the growth treatments, net photosynthesis was measured at saturating irradiance (1250 µmol m^–2^ s^–1^) at eleven CO_2_ concentrations between 50 and 1800 ppm. Measurements were made with a LI-6400 portable photosynthesis system (LI-COR) on one fully expanded, recently matured leaf per plant (eight plants per treatment) at growth temperature, and at 30 °C. VPD could not be maintained across measurement temperatures and averaged 1.0–1.3, 2.1–2.4, and 3.2–3.7 kPa at 25, 30, and 35 °C, respectively. These measurements enabled us to fit curves of *A* (assimilation rate) versus *c*_i_ (intercellular CO_2_ concentration). From these curves, we determined the maximum RuBP carboxylation rate (*V*_CMax_) and the maximum rate of RuBP regeneration (*J*_max_) according to the Farquhar, von Caemmerer and Berry model (FvCB) (Farquhar *et al.*, 1980; von Caemmerer and Farquhar, 1981) using the ‘Ecophys’ package (Duursma, 2015) in R version 3.1.0. The ‘Ecophys’ package uses temperature dependences for FvCB model parameters determined by Bernacchi *et al.* (2001). As a result of occasional non-convergence of curves in the analyses of *C. longifolium* leaves, means of *V*_CMax_ and *J*_Max_ for this species are based only on 4–7 replications per growth temperature instead of eight.

### Dark respiration

Dark respiration (*R*) was measured pre-dawn, first at growth temperature and then at 30 °C with an LI-6400 portable photosynthesis system at ambient relative humidity, 400 ppm CO_2_, and a flow rate of 300 µmol s^–1^. The same plants were studied as those used for *A*-*c*_i_ measurements. The temperature sensitivity of respiration was determined as the *Q*_10_, the proportional increase in respiration with a 10 °C increase in temperature. Respiration rates were standardized to 25 °C as:

$$R_{25}=\;\frac{R}{Q_{10}{}^{\left(0.1\times \left(T_{Leaf}-25\;\right)\right)}}$$ (3)

where *T*_Leaf_ was the leaf temperature as recorded by the leaf thermocouple of the LI-6400 cuvette and *Q*_10_ was set to 2.0 if it was not measured. Where respiration was measured at two temperatures, *Q*_10_ was calculated as:

$$Q_{10}={\left(\frac{R_{T_{1}}}{R_{T_{2}}}\right)}^{0.1\;\times \;\left(T_{1}-T_{2}\right)}$$ (4)

where $R_{T_{1}}$ and $R_{T_{1}}$ are the respiration rates at temperatures *T*_1_ and *T*_2_, respectively. Due to a technical issue, respiration could not be measured on *C. longifolium* plants.

### Growth analysis

At the end of the experiment, plants were harvested and dried at 70 °C for >96 h. Mean relative growth rate (RGR) was calculated using final mass (*M*_f_) of the measured plants and the average initial mass (*M*_i_) of three seedlings harvested at the start of the experiment as:

$$RGR=\;\frac{\text{ln}(M_{f})-\bar{\text{ln}(M_{i})}}{\Delta {\;}_{Time}}$$ (5)

### Statistical analyses

Differences in temperature-response traits across growth temperatures were analysed with ANOVA tests using Tukey *post hoc* tests. Correlations among traits were analysed as linear models. All analyses were performed in R, version 3.1.0 (R Development Core Team 2013, R Foundation for Statistical Computing, Vienna, Austria).

## Results

**Fig. 1.**
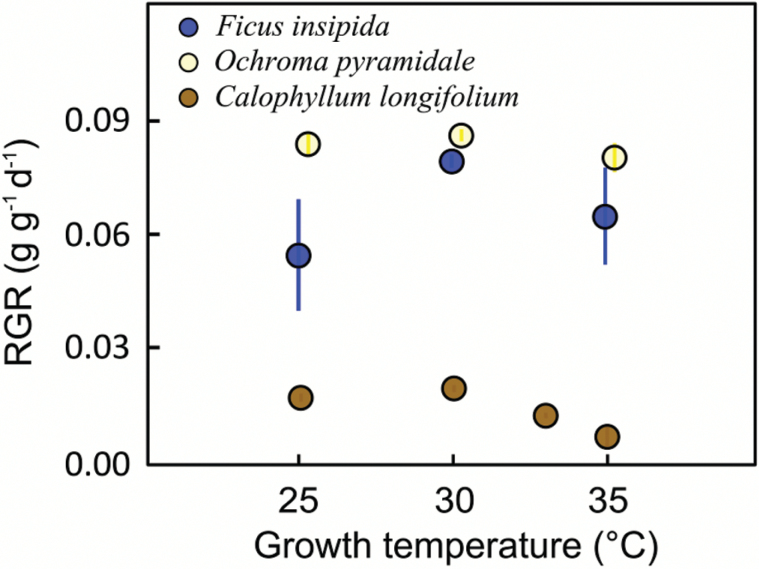
Mean relative growth rate (RGR) at different growth temperatures (±SE; *n*=4–8). The *x*-axis positions are slightly offset to improve visibility. (This figure is available in colour at *JXB* online.)

**Fig. 2.**
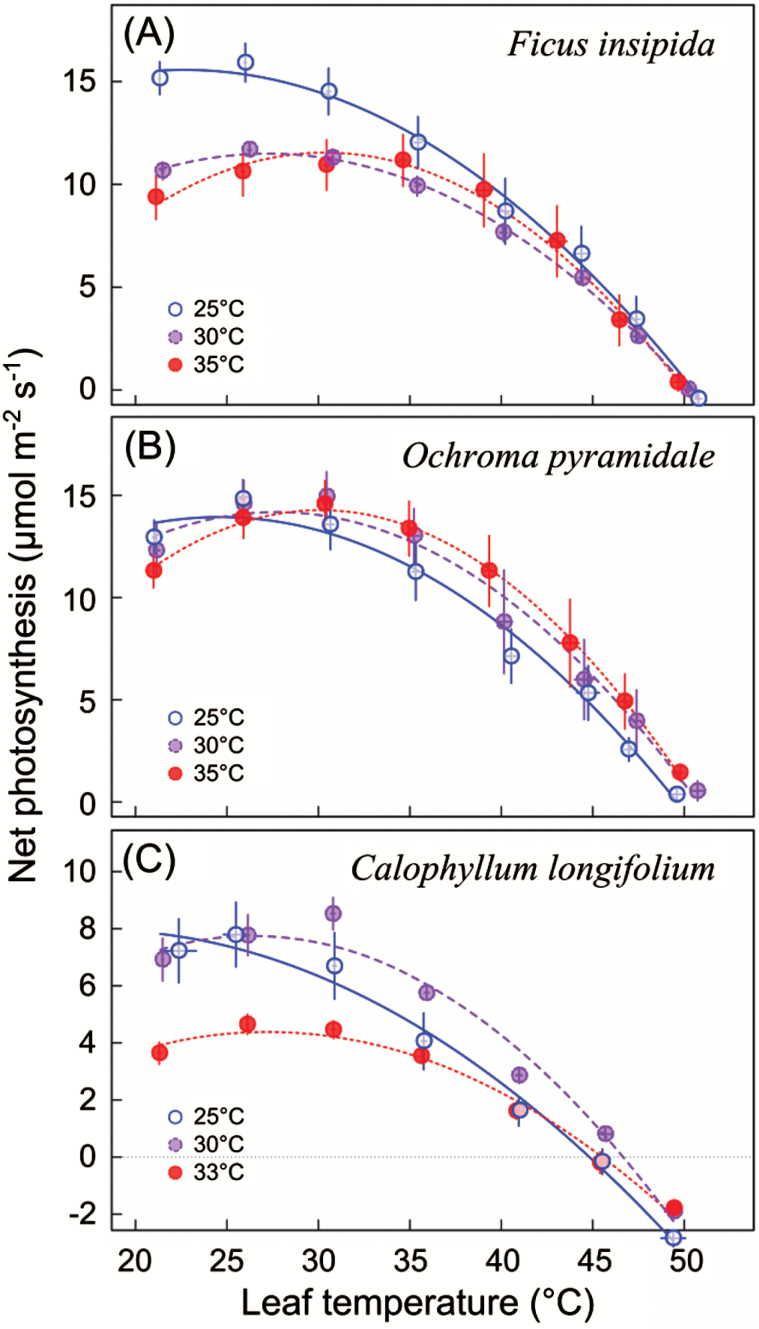
The temperature response of light-saturated photosynthesis in relation to growth temperature (means ±SE; *n*=4–6). Curves are fitted according to Eqn. 1. (This figure is available in colour at *JXB* online.)

**Fig. 3.**
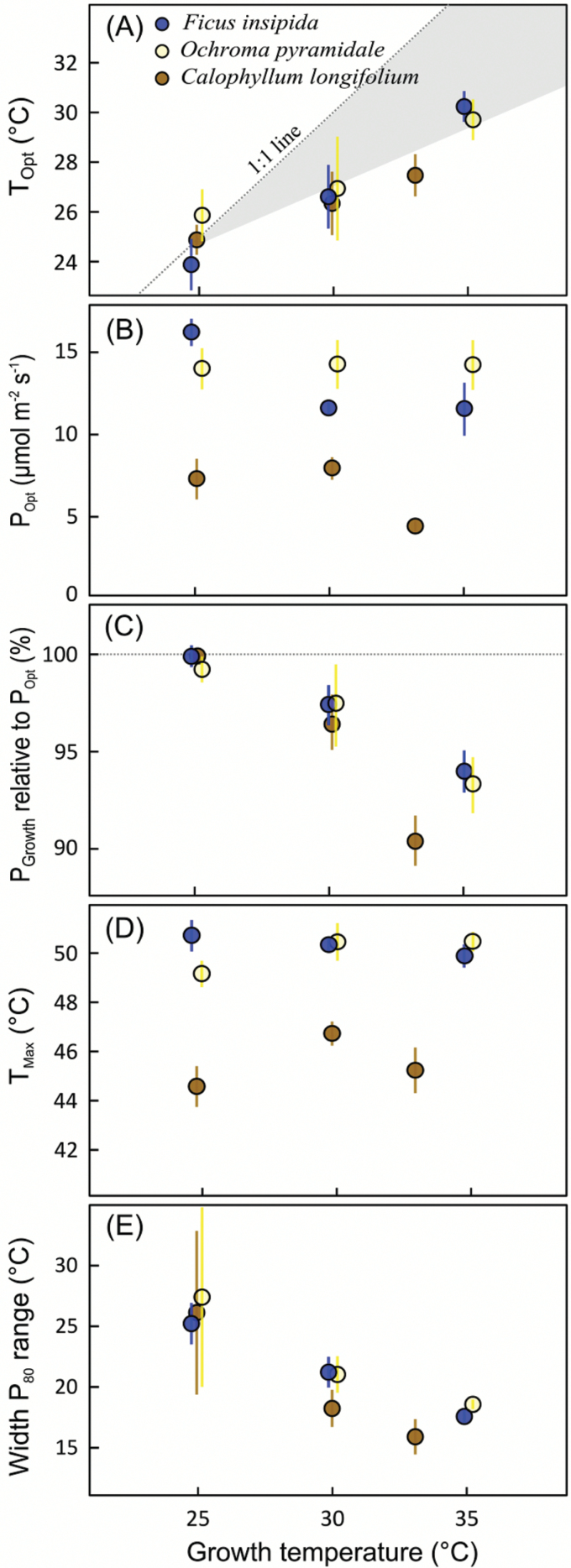
Temperature-response traits in relation to growth temperature (means ±SE; *n*=4–5). (A) Optimum temperature of photosynthesis (*T*_Opt_); (B) rate of photosynthesis at *T*_Opt_ (*P*_Opt_); (C) photosynthesis at growth temperature (*P*_Growth_) as a percentage of *P*_Opt_; (D) high-temperature CO_2_ compensation point (*T*_Max_); (E) width of the temperature range over which photosynthesis can perform at ≥80% of *P*_Opt_. The shaded area in (A) indicates the difference between *T*_Opt_ and *T*_Growth_. The large variance at 25 °C in (E) results from occasional under-estimation of *T*_Opt_ with Eqn. 1. The *x*-axis positions are slightly offset to improve visibility. (This figure is available in colour at *JXB* online.)

Temperature affected growth and appearance of all plants, with mean relative growth rate of all species peaking at the intermediate temperature of 30 °C (Fig. 1). Light-saturated photosynthesis in relation to measurement temperature could be fitted according to Eqn. 1 for all species (Fig. 2), with *R*^2^ values ranging from 0.89 to 0.99. The temperature-response curves peaked at higher temperature for plants grown at warmer conditions, i.e. *T*_Opt_ increased with growth temperature (Fig. 3A). *T*_Opt_ did not increase proportionally with growth temperature, resulting in an increasing discrepancy between the two. Across species, *T*_Opt_ increased by 0.47 °C per 1.0 °C increase in *T*_Growth_ and this regression curve intersects with the 1: 1 line at *T*_Growth_ of 24.3 °C; i.e. above 24.3 °C, *T*_Opt_ tends to be lower than *T*_Growth_ (Fig. 3A). It is clear from Fig. 2 that for plants grown at 25 °C, with only one measurement temperature below *T*_Opt_, the polynomial curve underestimated *T*_Opt_. We therefore also calculated *T*_Opt_ according to Eqn. 2, which avoids this issue, and *T*_Opt_ values calculated with the two methods are given in Table 1. Note that *T*_Opt_ values in Table 1 and Fig. 3 are the means of *T*_Opt_ values for replicate plants, whereas Fig. 2 shows temperature-response curves fitted to mean values of the replicate plants at different measurement temperatures.

**Table 1.** Photosynthesis temperature-response traits (means ±SE) of three tropical tree species grown at three different temperature regimes (*T*_Growth_)

**Species**	***T***_**Growth**_ **(°C)**	***T***_**Opt**_ **(°C)**		***P***_**Opt**_ **(µmol m** ^**–2**^ **s** ^**–1**^)	***P***_**Growth**_ **(µmol m** ^**–2**^ **s** ^**–1**^)	***T***_**Max**_ **(°C)**	**Width *P***_**80**_ **range (°C**)	***V***_**CMax**_ **(µmol m** ^**–2**^ **s** ^**–1**^)	***J***_**Max**_ **(µmol m** ^**–2**^ **s** ^**–1**^)
		**Eqn. 1**	**Eqn. 2**						
*Ficus insipida*	25	23.9 ± 1.0	26.2 ± 0.5	16.3 ± 0.8	16.2 ± 0.8	50.7 ± 0.6	25.2 ± 1.7	69 ± 2	159 ± 5
	30	26.6 ± 1.3	28.0 ± 0.7	11.6 ± 0.4	11.3 ± 0.3	50.3 ± 0.3	21.2 ± 1.3	95 ± 8	160 ± 5
	35	30.2 ± 0.6	30.2 ± 0.6	11.6 ± 1.6	10.9 ± 1.4	49.9 ± 0.5	17.6 ± 0.5	93 ± 5	128 ± 10
*Ochroma pyramidale*	25	25.9 ± 1.0	26.7 ± 1.1	14.1 ± 1.3	13.9 ± 1.4	49.2 ± 0.5	27.4 ± 7.4	79 ± 3	172 ± 4
	30	26.9 ± 2.1	28.9 ± 0.5	14.3 ± 1.5	14.0 ± 1.5	50.5 ± 0.8	21.0 ± 1.5	122 ± 4	176 ± 5
	35	29.7 ± 0.8	29.9 ± 0.6	14.3 ± 1.5	13.4 ± 1.5	50.5 ± 0.4	18.6 ± 0.4	181 ± 11	212 ± 10
*Calophyllum longifolium*	25	24.9 ± 0.6	26.2 ± 0.5	7.3 ± 1.2	7.3 ± 1.4	44.6 ± 0.8	26.1 ± 6.7	40 ± 2	72 ± 7
	30	26.3 ± 1.3	28.1 ± 0.5	8.0 ± 0.7	7.7 ± 0.5	46.7 ± 0.5	18.2 ± 1.5	70 ± 17	94 ± 17
	33	27.5 ± 0.9	28.1 ± 0.2	4.4 ± 0.3	4.0 ± 0.3	45.2 ± 0.9	15.9 ± 1.4	68 ± 8	66 ± 5

*T*_Opt_, optimum temperature of photosynthesis calculated using Eqn. 1 and Eqn. 2; *P*_Opt_, the rate of photosynthesis at *T*_Opt_; *P*_Growth_, the rate of photosynthesis at growth temperature (*T*_Growth_); *T*_Max,_ the high-temperature CO_2_ compensation point; *P*_80_, the width of the temperature range over which plants operate at ≥80% of *P*_Opt_; *V*_CMax_, the maximum rate of RuBP carboxylation; *J*_Max_, the maximum rate of RuBP regeneration.

The rate of photosynthesis at *T*_Opt_ (*P*_Opt_) did not change systematically with growth temperature (Fig. 3B), regardless of whether *T*_Opt_ was calculated using Eqn. 1 or Eqn. 2. For *O. pyramidale* it did not change at all, whereas for *F. insipida* rates at 30 and 35 °C were significantly lower than at 25 °C (*P*=0.006 and *P*=0.01, respectively), and for *C. longifolium P*_Opt_ was marginally reduced at 33 compared to 25 °C (*P*=0.07; ANOVA with Tukey *post hoc* tests). Photosynthesis rates at growth temperature (*P*_Growth_), calculated from Eqn. 1, decreased relative to *P*_Opt_ with increasing growth temperature (Table 1), with *P*_Growth_ equaling *P*_Opt_ at 25 °C, but decreasing by 6–10% at the highest growth temperature (Fig. 3C). The high-temperature CO_2_ compensation point, or *T*_Max_, was not affected by growth temperature (Fig. 2), but there was a significant species effect (ANOVA, *P*<0.001), because *C. longifolium* had lower *T*_Max_ than the early-successional species *F. insipida* and *O. pyramidale* at each growth temperature (Fig. 3D). As a result of an increase in *T*_Opt_ and no change in *T*_Max_, the curves narrowed with increasing growth temperature, as illustrated by a decrease in the width of the temperature range over which plants can operate at 80% or more of their *P*_Opt_, as calculated from the curves generated with Eqn 1 (Fig. 3E).

**Fig. 4.**
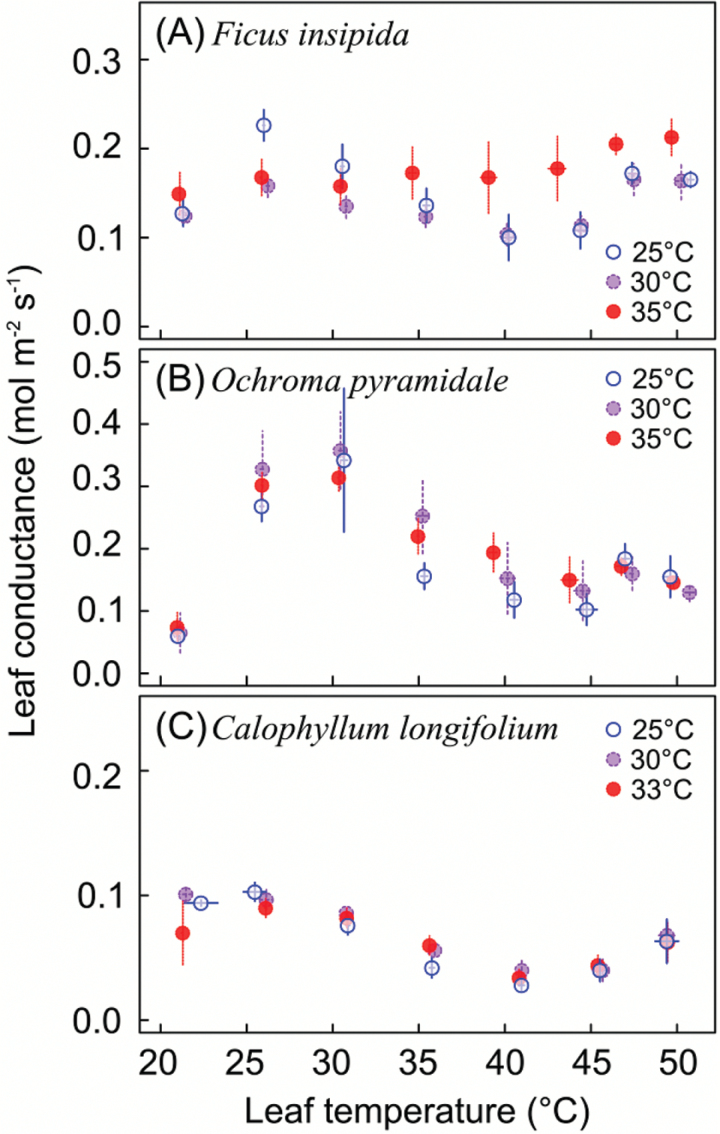
Leaf conductance in relation to leaf temperature for seedlings grown at different temperatures (means ±SE; *n*=4–8). (This figure is available in colour at *JXB* online.)

**Fig. 5.**
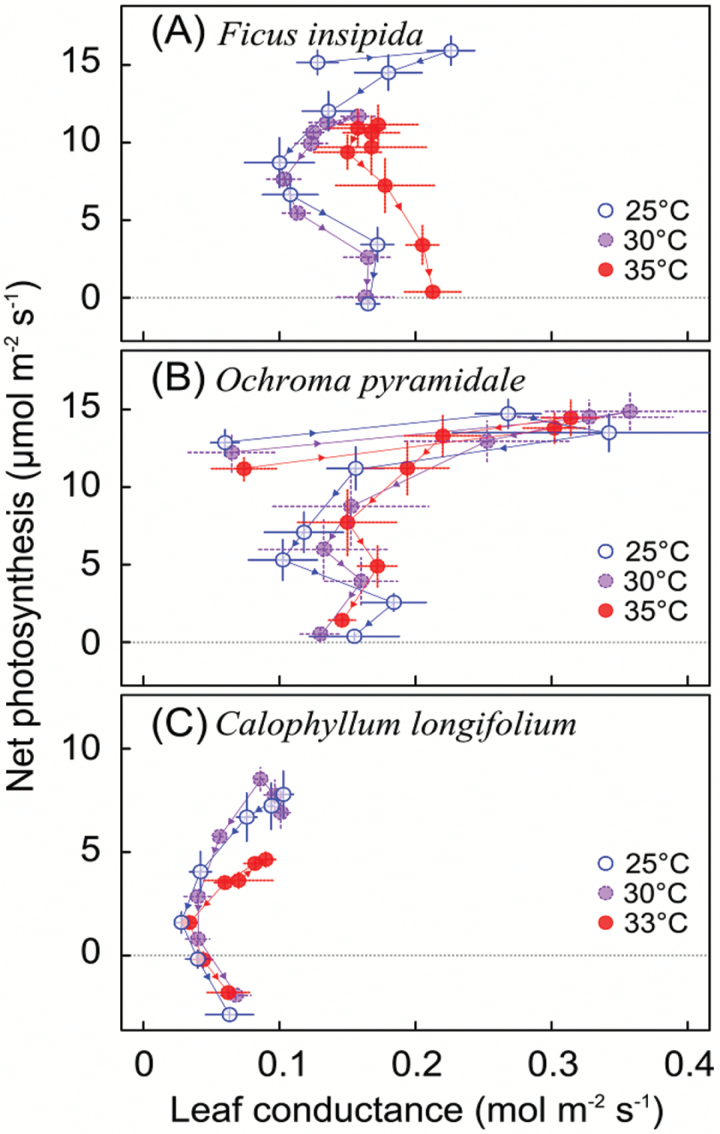
Photosynthesis in relation to leaf conductance for seedlings grown at different temperatures (means ±SE; *n*=4–8). Lines and arrows indicate the directional pattern from the lowest to highest measurement temperature. (This figure is available in colour at *JXB* online.)

Leaf conductance to water vapor transfer generally peaked between 25 and 30 °C, before gradually dropping off at higher temperatures (Fig. 4). However, when temperatures exceeded ~40 °C, conductance increased again. These temperature-response patterns of leaf conductance were consistent across species and growth temperatures. Only in *F. insipida* was conductance above growth temperature higher for plants grown at 35 °C than for those grown at 25 or 30°C (Fig. 4). Because the temperature response of leaf conductance was not unimodal like photosynthesis, and conductance increased when temperatures approached *T*_Max_, photosynthesis did not correlate strongly with conductance across leaf temperatures (Fig. 5).

**Fig. 6.**
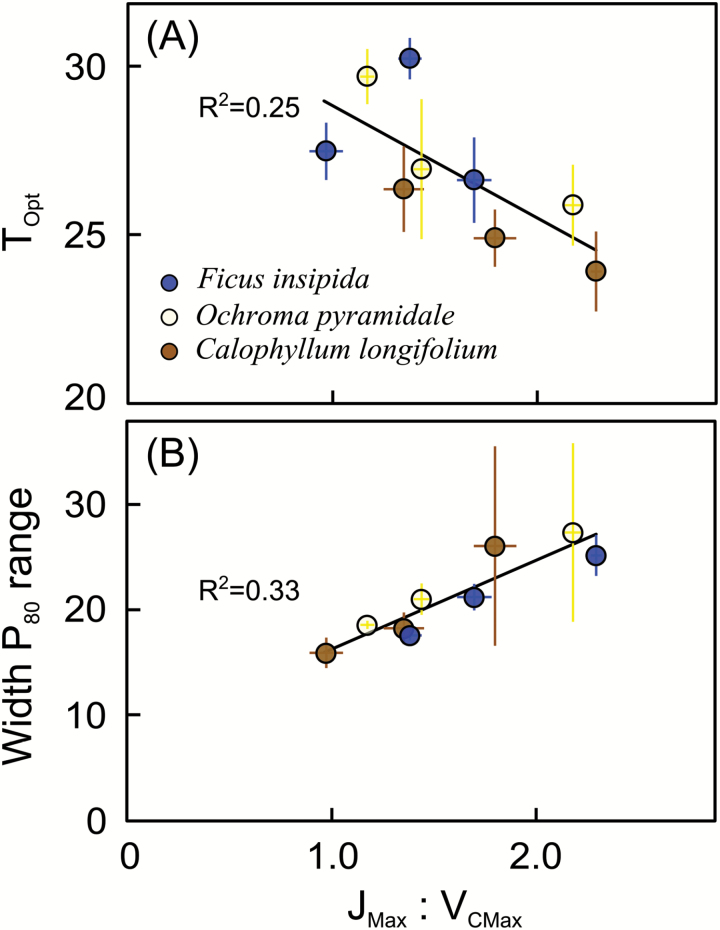
(A) The optimum temperature for photosynthesis (*T*_Opt_) and (B) the width of the temperature range over which photosynthesis can operate at 80% or more of the rate at *T*_Opt_ in relation to the ratio of the maximum rates of RuBP regeneration and RuBP carboxylation (*J*_Max_:*V*_CMax_). For each species, mean values per growth temperature are shown (±SE; *n*=8 for *F. insipida* and *O. pyramidale*, *n*=4–7 for *C. longifolium*). Black lines indicate significant correlations of plant-level data, with *P*=0.002 in (A), and *P*<0.001 in (B). (This figure is available in colour at *JXB* online.)

*V*_CMax_ increased with measurement temperature, but when all plants were measured at the same temperature (30 °C) it did not differ consistently among plants grown at different temperature regimes (data not shown). Consequently, when measured at growth temperature, *V*_CMax_ increased with temperature across species (*R*^2^=0.38, *P*<0.001) (see also Table 1). *J*_Max_ similarly increased with measurement temperature, but it did not systematically increase with temperature when plants were measured at their respective growth temperatures (*R*^2^=0.02, *P*=0.38) (Table 1). The ratio of *J*_Max_ to *V*_CMax_ therefore decreased with increasing growth temperature (*R*^2^=0.69, *P*<0.001). Across species and growth temperatures, *T*_Opt_ correlated negatively with the ratio of *J*_Max_ to *V*_CMax_ (Fig. 6A), and within species this correlation was also significant for *O. pyramidale* and *C. longifolium*. These correlations were even stronger and significant for all species when *T*_Opt_ values were calculated using Eqn. 2 (data not shown). Plants that had a high *J*_Max_ to *V*_CMax_ ratio also had a wide *P*_80_ temperature range (Fig. 6B).

**Fig. 7.**
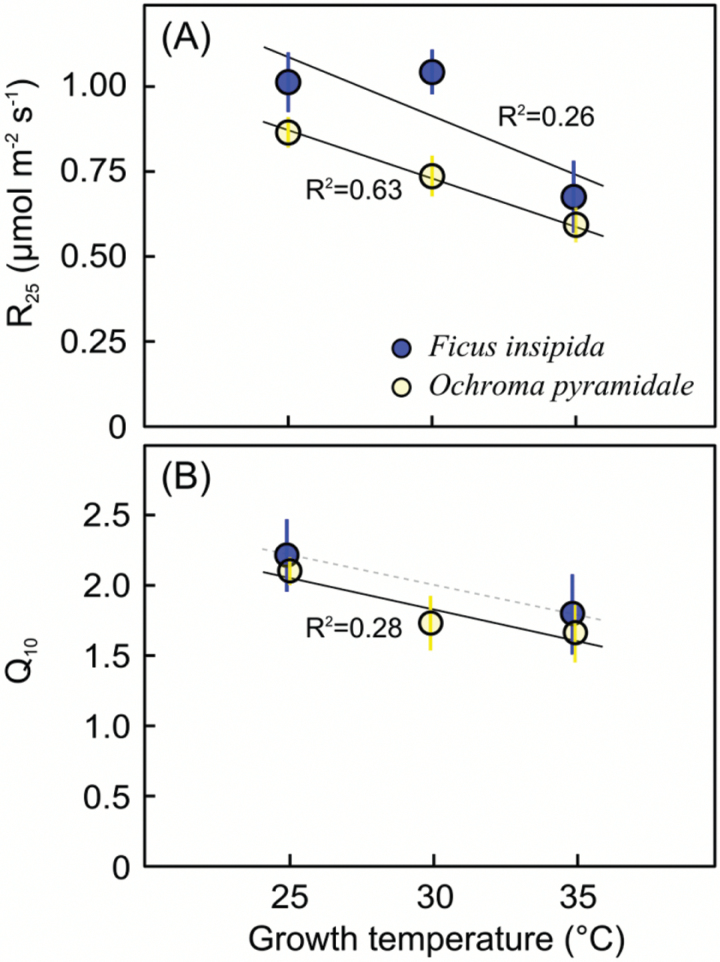
(A) Dark respiration standardized to 25 °C (*R*_25_) and (B) the temperature sensitivity of dark respiration (*Q*_10_) in relation to growth temperature (means ±SE). Solid black lines indicate significant correlations of plant-level data, with *P*=0.012 and *P*<0.001 for *F. insipida* and *O. pyramidale*, respectively, in (A), and *P*=0.033 for *O. pyramidale* in (B). The *x*-axis positions are slightly offset to improve visibility. (This figure is available in colour at *JXB* online.)

Dark respiration rates standardized at 25 °C (*R*_25_) significantly decreased with increasing growth temperature in both *F. insipida* (*P*=0.012) and *O. pyramidale* (*P*<0.01) (Fig. 7A). The temperature-sensitivity of dark respiration, expressed by the *Q*_10_, decreased significantly with growth temperature only in *O. pyramidale* (*P*=0.032) (Fig. 7B).

Leaf carbon and nitrogen concentrations did not change systematically with increasing growth temperature, although there was a positive correlation between leaf N and growth temperature in *O. pyramidale* (Supplementary Table S1 at *JXB* online). Leaf mass per unit leaf area (LMA) decreased with increasing growth temperature in all species, causing C per unit leaf area to decrease with increasing growth temperature in all species.

## Discussion

We have shown that tropical tree species have the capacity to thermally acclimate to elevated growth temperatures. Acclimation was expressed by a shift in optimum temperature and no systematic change in photosynthesis rate at optimum temperature. The shift in optimum temperature of photosynthesis, and the down-regulation of dark respiration, improve carbon gain at elevated temperatures. Acclimation was not perfect, however, in the sense that as growth temperature increased, the temperature-response curves narrowed, and the position of their peak, *T*_Opt_, did not increase as much as *T*_Growth_. Therefore, as temperatures increased, plants increasingly operated at temperatures that were supra-optimal to photosynthesis, resulting in less carbon uptake than perfectly acclimated plants would have been able to achieve. This observation is consistent with a global meta-analysis by Yamori *et al.* (2014), which showed that *T*_Opt_ of C_3_ species only increases by, on average, 0.5 °C for every 1.0 °C increase in *T*_Growth_, with *T*_Opt_ < *T*_Growth_ when *T*_Growth_ >26 °C.

As growth temperature increases, so does VPD, which in well-watered plants can lead to increased transpiration rates and thus to increased leaf cooling. Indeed, snapshot measurements of leaf temperature of *C. longifolium* and *O. pyramidale* with an infrared thermometer (MiniTemp MT6, Raytek, Santa Cruz, CA, USA) showed that leaf temperatures were maintained slightly above air temperature in plants grown at 25 °C, but at 35 °C leaves of the early-successional species *O. pyramidale* were cooled by ~1–2 °C, while the late-successional *C. longifolium* leaves tracked air temperature. The shift in tissue temperature relative to air temperature reduces but does not eliminate the growing gap between growth temperature and *T*_Opt_ of net photosynthesis with warming.

### High-temperature increase in leaf conductance

Stomatal conductance plays an important role in the reduction of net photosynthesis above *T*_Opt_ (e.g. Lin *et al.*, 2012; Slot and Winter, 2017*a*), but differences in photosynthetic characteristics between plants grown at contrasting temperature regimes cannot be explained by stomatal responses alone (Berry and Björkman, 1980). Indeed, at a given leaf temperature, stomatal conductance did not differ between different growth temperatures, with the exception of a few measurements of *F. insipida* seedlings grown at 35 °C. Interestingly, however, and consistent with Slot *et al.* (2016), we found a distinct rise in conductance to water vapor transfer at temperatures >40 °C, a phenomenon that we have never observed in the field (Slot *et al.*, 2016; Slot and Winter, 2017*a*). The field measurements concerned canopy trees, and it is possible that in contrast to potted seedlings, hydraulic constraints limit the delivery of water to the upper canopy under high temperature/high VPD conditions. As the temperatures in the cuvette approach critical levels that lead to irreversible leaf damage and necrosis, transpirational cooling is certainly highly advantageous. Indeed, above air temperatures of 40 °C the degree of leaf cooling below the set temperature rapidly increased, resulting in leaf temperatures ~5 °C below air temperatures when the air temperature was 55 °C. It is also possible that the observed increase in conductance did not reflect stomatal opening, but a change in permeability of leaf cuticles, which commonly increases steeply above 40 °C as a result of thermal expansion of the cuticular polymers (Riederer, 2006). Leaves in the field typically experience temperatures >40 °C for very short duration, and as such may not experience the changes in cuticular properties that may cause the increase in water vapor transfer conductance under laboratory conditions.

### Thermal acclimation, models, and biochemical parameters

Down-regulation of dark respiration in plants grown at high temperature and the decrease in *Q*_10_ are consistent with thermal acclimation (Atkin and Tjoelker, 2003), and confirm previous observations on tropical tree species (Cheesman and Winter, 2013; Slot *et al.*, 2014). Acclimation of respiration is now recognized as a global phenomenon (Slot and Kitajima, 2015; Vanderwel *et al.*, 2015), but global models still largely ignore acclimation (Smith and Dukes, 2013; Lombardozzi *et al.*, 2015), in part because of the paucity of empirical data required for parameterization of acclimation algorithms. Thermal acclimation of photosynthetic parameters has been described in detailed algorithms (e.g. Bernacchi *et al.*, 2003; Hikosaka *et al.*, 2006; Kattge and Knorr, 2007), but information for tropical forest species is scarce (e.g. Lombardozzi *et al.*, 2015), and global vegetation models either do not account for photosynthetic acclimation, or, if they do, they assume that acclimation occurs similarly in all plant functional types and in all biomes.

Vegetation models generally do not use net photosynthesis, but instead use *V*_CMax_, the maximum RuBP carboxylation capacity, and *J*_Max_, the maximum rate of RuBP regeneration, which is generally assumed to reflect the maximum electron transport rate. In our study, *V*_CMax_ changed more strongly with growth temperature than *J*_Max_. As a result, warm-grown plants had lower *J*_Max_:*V*_CMax_ ratios, consistent with studies of cooler-climate vegetation (Bernacchi *et al.*, 2003; June *et al.*, 2004; Hikosaka *et al.*, 2006; Kattge and Knorr, 2007; Dusenge *et al.*, 2015). This change in the *J*_Max_:*V*_CMax_ ratio probably reflects changes in the allocation of photosynthetic proteins (Sung *et al.*, 2003; Yamori *et al.*, 2005). The *J*_Max_:*V*_CMax_ ratio correlated strongly and negatively with *T*_Opt_, suggesting that the acclimation of *T*_Opt_ was a result of *J*_Max_ exerting greater control over light-saturated photosynthesis than in plants grown at lower temperatures – and that RuBP-regeneration-limited photosynthesis had a higher temperature optimum than RuBP-carboxylation-limited photosynthesis, *V*_CMax_, consistent with the mechanism proposed by Hikosaka *et al.* (2006). Recent observations for montane tropical tree species, however, found a lower *T*_Opt_ of *J*_Max_ than of *V*_CMax_ and electron-transport limitation of photosynthesis at high measurement temperatures (Vårhammar *et al.*, 2015). Clearly, more detailed measurements of *A*-*c*_i_ curves across a wide temperature range are needed for lowland tropical forest trees to better understand the biochemical adjustments underlying the observed acclimation response.

Another component of acclimation of net photosynthesis is the degree to which respiration in the light (non-photorespiratory mitochondrial respiration) acclimates (Way and Yamori, 2014). Respiration in the light tends to be lower than in the dark but, as with dark respiration, it increases with temperature and may acclimate to warming by down-regulation at elevated growth temperatures (e.g. Way and Sage, 2008). Especially at high temperatures and low gross photosynthesis, the degree of acclimatory down-regulation of respiration in the light could influence the observed temperature-response of net photosynthesis (Way and Yamori, 2014). Respiration in the light is generally strongly correlated with respiration in the dark (e.g. Atkin *et al.*, 2006; Ayub *et al.*, 2011), and since respiration in the dark was down-regulated in warm-grown plants, part of the observed acclimation in net photosynthesis probably resulted from changes in respiration in the light.

Scafaro *et al.* (2017) recently showed that thermal acclimation of photosynthesis of Australian tree species was, in part, underpinned by changes in leaf Rubisco content. As a proxy for Rubisco content, we analysed leaf nitrogen content, but we did not find consistent patterns with growth temperature, probably because photosynthesis at optimum temperature did not change systematically, as it did for most species in Scafaro *et al.* (2017).

To improve the representation of thermal acclimation in vegetation- and Earth System models, the biochemical as well as the stomatal temperature dependences underlying the temperature response of net photosynthesis will need to be quantified for a diverse set of tropical species representative of different plant functional types. In addition to the temperature responses of *V*_CMax_ and *J*_Max_, the temperature dependences of Rubisco activase, Rubisco kinetics, mesophyll conductance, and respiration in the light would need to be determined (Busch and Sage, 2017; Rogers *et al.*, 2017).

### Temperature-response curves narrow with increasing growth temperature

The *J*_Max_:*V*_CMax_ ratio correlated with *T*_Opt_ and, because temperature-response curves contracted as *T*_Opt_ increased with increasing growth temperature, *J*_Max_:*V*_CMax_ also correlated strongly with the width of the temperature range over which plants can operate at 80% or more of their maximum photosynthetic capacity. Curve contraction at higher growth temperatures was caused by inflexibility of *T*_Max_, the high-temperature CO_2_ compensation point. For the two early-successional species, *T*_Max_ approached temperatures known to induce irreversible damage to leaves (e.g. Krause *et al.*, 2010, 2013), and as such these *T*_Max_ values are likely to reflect absolute thermal limits. O’Sullivan *et al.* (2017) recently showed that the maximum temperature of PSII integrity was higher in the tropics (50.8 °C) than in high latitudes (41.5 °C in Alaska), but so far there is no experimental confirmation that high growth temperature can increase heat tolerance markedly above ~51 °C (Krause *et al.*, 2013), with the exception of CAM succulents (Krause *et al.*, 2016).

### Acclimation and tropical forests in a warming world

Acclimation significantly reduces the potential negative effects of high temperature on net carbon uptake of tropical forest trees. As temperature continues to rise, however, the difference between *T*_Opt_ and ambient temperature grows, reducing net photosynthesis at growth temperature relative to photosynthesis at optimum temperature. As temperature-response curves continue to narrow, more time is spent at temperatures too high for optimal photosynthetic performance. Furthermore, while the curves reported here are fairly wide, we know from field observations that tropical canopy trees have *T*_Max_ values in the range of 39–46 °C (Slot *et al.*, 2016; Slot and Winter, 2017*a*), and thus the temperature range over which a net carbon uptake can be maintained is narrower for canopy trees in the field than reported here. Photosynthesis of tropical forest trees peaks at current ambient temperatures (Slot and Winter, 2017*a*). Acclimation to moderate warming should enable tropical trees to continue to optimize photosynthetic carbon uptake in the near future. However, incomplete acclimation to more extreme warming will result in decreased net carbon uptake as plants more often experience temperatures above *T*_Opt_. If incomplete acclimation is the norm in tropical forests this could have major consequences for the global carbon cycle. However, whether changes in photosynthesis translate into changes in plant growth and forest carbon storage will depend on the extent to which elevated [CO_2_] ameliorates the high-temperature effects on tropical species, and on the extent to which tropical forest growth is carbon limited (Lloyd and Farquhar, 2008;Körner, 2009), and in the long term it will also depend on demographic changes within the tree community. Relative growth rate of field-grown tropical saplings has been shown not to be affected by moderate warming, while combined warming and elevated [CO_2_] may stimulate sapling growth (K. Winter *et al*., unpublished results). Clearly, more detailed analyses will be needed to link the capacity for thermal acclimation of photosynthesis to future tree growth in tropical forests.

## Supplementary data

Supplementary data can be found at *JXB* online.

Table S1. Leaf chemical and morphological traits of three tropical tree species grown at three different temperature regimes (*T*_Growth_).

## Data deposition

Data from all three species and growth temperatures on temperature-response of photosynthesis, respiration at contrasting temperature, *J*_Max_ and *V*_CMax_, and relative growth rate are available at Dryad Digital Repository. doi:10.5061/dryad.r11c0.

## Supplementary Material

supplementary_table_S1

## Abbreviations:

A: assimilation rate (µmol m^–2^ s^–1^)
*c*_i_: intercellular CO_2_ concentration (µmol mol^–1^)
*J*_Max_: maximum rate of RuBP regeneration (µmol m^–2^ s^–1^)
*P*_Growth_: photosynthesis rate at growth temperature (µmol m^–2^ s^–1^)
*P*_Opt_: photosynthesis rate at optimum temperature (µmol m^–2^ s^–1^)
PSII: photosystem II
*Q*_10_: proportional increase in dark respiration with 10 °C increase in temperature
R: dark respiration rate (µmol m^–2^ s^–1^)
*R*_25_: dark respiration rate at 25 °C (µmol m^–2^ s^–1^)
RGR: relative growth rate (g g^–1^ d^–1^)
*T*_Leaf_: leaf temperature (°C)
*T*_Max_: high-temperature CO_2_ compensation point (°C)
*T*_Opt_: optimum temperature for light-saturated photosynthesis (°C)
*V*_CMax_: maximum rate of RuBP carboxylation (µmol m^–2^ s^–1^)
VPD: vapor pressure deficit (kPa).
