# Supplementary material for: Photosynthetic acclimation to warming in tropical forest tree seedlings
Source: J Exp Bot. 2017 Apr 27;68(9):2275–84. doi: 10.1093/jxb/erx071 (PMC5447879; doi:10.1093/jxb/erx071)
Supplement: supplementary_table_S1 [file erx071_suppl_supplementary_table_S1.pdf]

Photosynthetic acclimation to warming in tropical forest tree seedlings

Martijn Slot and Klaus Winter

Supplementary data

Table S1. Leaf chemical and morphological traits (means ± standard errors) of three tropical tree species grown at three different temperature regimes (T_{Growth}).

**Species**	**T_{Growth}** °C	**N** mg g^{-1}	**N_{Area}** g m^{-2}	**C** mg g^{-1}	**C_{Area}** g m^{-2}	**C : N ratio**	**LMA** g m^{-2}
Ficus	25	29.2 ± 1.1	1.64 ± 0.13	392 ± 2	21.6 ± 0.9	13.5 ± 0.5	55.2 ± 2.3
insipida	30	29.8 ± 1.4	1.34 ± 0.08	395 ± 2	18.5 ± 0.6	13.4 ± 0.6	47.1 ± 1.4
	35	21.1 ± 2.1	0.95 ± 0.07	392 ± 3	16.8 ± 0.8	19.0 ± 1.8	42.9 ± 2.4
Ochroma	25	21.0 ± 3.2	1.41 ± 0.30	424 ± 4	23.2 ± 0.6	21.8 ± 2.7	54.0 ± 1.1
pyramidale	30	25.9 ± 2.3	1.24 ± 0.08	431 ± 3	21.1 ± 1.0	17.4 ± 1.6	48.9 ± 2.2
	35	34.3 ± 3.7	1.35 ± 0.11	415 ± 2	16.6 ± 1.0	12.7 ± 1.7	40.0 ± 2.5
Calophyllum	25	12.6 ± 0.9	1.66 ± 0.14	461 ± 2	61.3 ± 4.2	37.3 ± 2.7	133.2 ± 9.5
longifolium	30	16.8 ± 1.1	2.00 ± 0.16	465 ± 2	55.4 ± 1.7	28.1 ± 1.6	119.1 ± 3.4
	33	14.6 ± 0.8	1.55 ± 0.08	461 ± 3	48.0 ± 3.8	31.8 ± 1.8	103.3 ± 7.7

Shown are leaf nitrogen (N) and carbon (C) content on a leaf mass basis and on an area basis (subscript “Area”), their ratio (C : N ratio) and the leaf mass per unit leaf area (LMA). Values are means ± standard errors (n=4–7).
